# Risk factors for progression to bronchitis or pneumonia in hospitalized children with human rhinovirus infection: a retrospective cohort study using machine learning

**DOI:** 10.3389/fped.2026.1787440

**Published:** 2026-04-14

**Authors:** Qiaoyan Dai, Jingying Zhou, Ying Chen, Qidong Ye

**Affiliations:** Department of Pediatrics, The First Affiliated Hospital of Ningbo University, Ningbo, China

**Keywords:** bronchitis, machine learning, pediatric infectious diseases, pneumonia, rhinovirus

## Abstract

**Background:**

Human rhinovirus (HRV) is a leading cause of acute respiratory infections and hospitalization in children. However, risk factors for progression to bronchitis vs. pneumonia remain incompletely characterized.

**Objective:**

This study aimed to identify and distinguish independent predictors for these outcomes using a machine learning approach.

**Methods:**

A retrospective cohort study was conducted among hospitalized children with HRV infection at the First Affiliated Hospital of Ningbo University. A two-stage feature selection method (Random Forest and LASSO) was used, followed by multinomial logistic regression to quantify associations with progression to bronchitis or pneumonia.

**Results:**

Of 1,125 children, 29% progressed to bronchitis and 59% to pneumonia. Multinomial logistic regression revealed distinct risk profiles. Progression to bronchitis was strongly associated with lower airway obstruction, including dyspnea (*OR:* 4.35, 95% *CI*: 2.35–8.06), wheezing on auscultation (*OR:* 2.98, 95% *CI*: 1.89–4.69), and cough (*OR:* 2.61, 95% *CI*: 1.52–4.49). Progression to pneumonia was uniquely linked to prior antibiotic use (*OR:* 2.09, 95% *CI*: 1.34–3.25), viral co-infection (*OR:* 1.97, 95% *CI*: 1.25–3.10), and coagulation abnormalities (*OR:* 1.04, 95% *CI*: 1.00–1.08). Elevated IgM was a common risk factor for both, while older age was protective against both (*OR:* 0.89, 95% *CI*: 0.83–0.96).

**Conclusion:**

Bronchitis progression is primarily associated with airway obstruction, whereas pneumonia is linked to more complex clinical scenarios, including prior medication, co-infections, and coagulopathy. These findings can improve early risk stratification and guide targeted interventions.

## Introduction

Human rhinovirus (HRV) is a very common respiratory virus, especially in children (1, 2). It is commonly associated with mild illnesses such as the common cold, but can also lead to more severe outcomes including lower respiratory tract infections (LRTIs) like bronchitis and pneumonia (3–5). These LRTIs represent a major cause of hospitalization and significant morbidity in children (6–8). Moreover, HRV-associated bronchitis or pneumonia in early childhood may contribute to the development of chronic asthma (9, 10). Early identification of children at risk of progression from mild HRV infection to LRTIs is essential for improving clinical outcomes and reducing long-term complications.

Most previous clinical studies on HRV infection in children have focused on high-risk populations, such as those with allergic rhinitis (11), bronchopulmonary dysplasia (12), or organic transplantation (13, 14). These studies have shown that HRV infection in high-risk children can lead to increased disease severity, exacerbation of respiratory symptoms, and triggering of wheezing or asthma attacks. However, evidence is emerging that early-life HRV infection may also matter in children without known risk factors. A low-risk birth infant cohort found that HRV-induced wheezing during the first year of life predicted subsequent wheezing and physician-diagnosed asthma equally in both high- and low-risk children (15). However, it still remains unknow that how healthy children progress from mild HRV infection to acute LRTIs (16, 17). Large studies examining this progression in the general pediatric population are still scarce.

Recent studies have identified young age, bacterial co-infection, immune dysfunction, and certain clinical symptoms for disease progression in children with HRV infection (18, 19). A study among 34 children with allergic rhinitis found that elevated levels of nasal IgE, interleukin-25 (IL-25), IL-4, and CXCL13 were associated with worsening of upper respiratory symptoms (11). Another study on 178 patients suggested that viral genotype and viral load may also influence disease severity (20). Nevertheless, most of these studies were limited by small sample sizes and cross-sectional design.

Therefore, this study aims to identify key baseline determinants, including pre-admission clinical factors and laboratory indicators collected within 24 h of hospital admission, for the progression of HRV infection to bronchitis or pneumonia in hospitalized children. Machine learning-based feature selection was integrated with multinomial logistic regression to construct a quantitative model for predicting individual risk and guiding early clinical intervention.

## Materrials and methods

### Study design and participants

**Figure 1:**
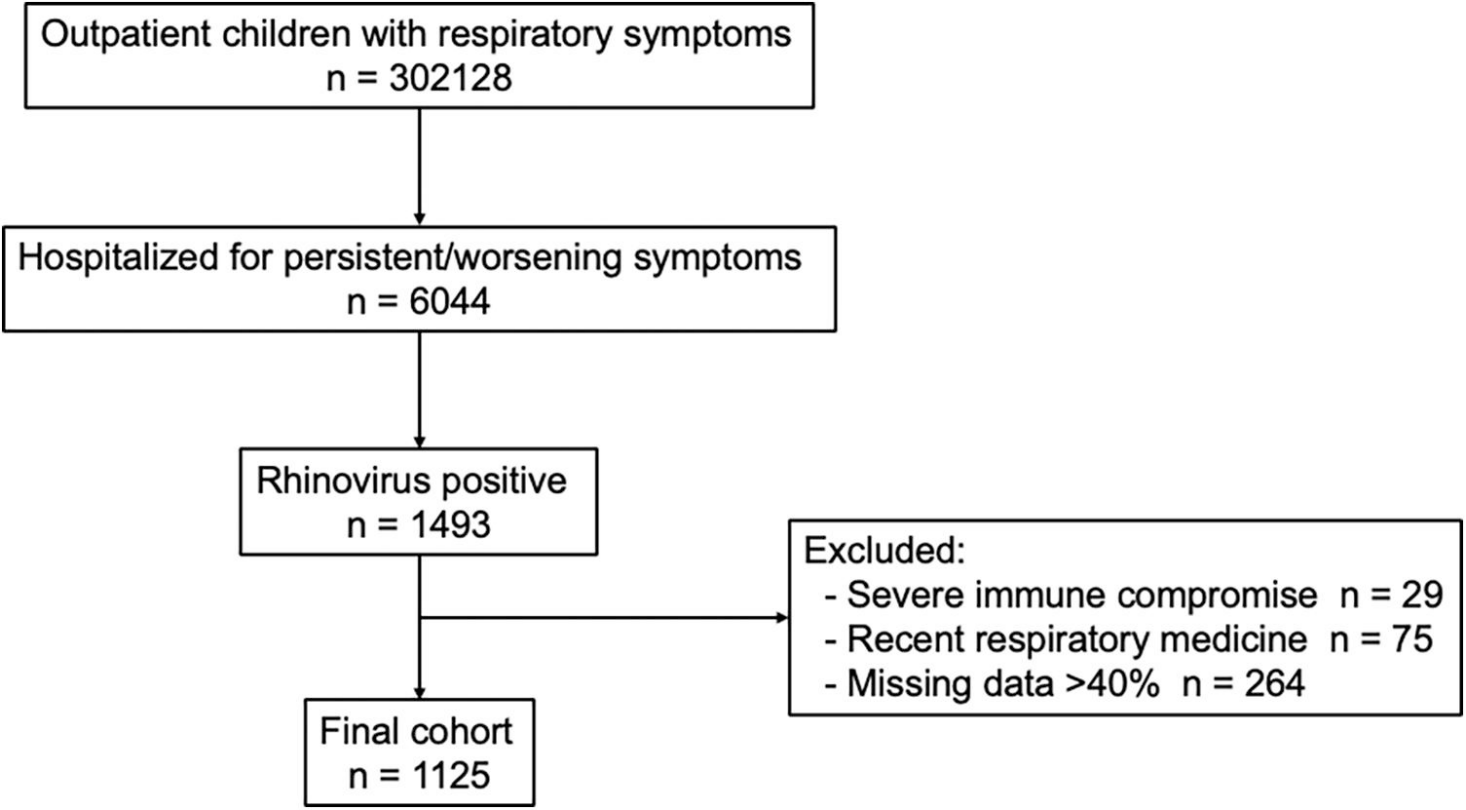
Flowchart of participant selection in the retrospective cohort of hospitalized children with rhinovirus infection.

A single-center, retrospective cohort study was conducted at the First Affiliated Hospital of Ningbo University. The study period was from February 2023 to January 2025. The study population consisted of children aged 0–14 years who had previously been seen in the outpatient department for acute respiratory symptoms and required hospital admission due to persistent high fever (typically ≥3–5 days), significant cough or respiratory symptoms, young age requiring close observation, or clinical concern for potential progression to lower respiratory tract infection. In our hospital, multiplex PCR testing for respiratory viruses is routinely performed for all children admitted with suspected acute respiratory tract infections. Nasopharyngeal-swab multiplex PCR testing was performed within 24 h of admission. Following admission, children were monitored daily for the development of lower respiratory tract involvement. This approach allows assessment of disease progression over time. Children were included if they had a positive rhinovirus result on multiplex PCR. Children with severe immune-compromising conditions, including primary immunodeficiency, hematological malignancy, or solid-organ transplantation, were excluded. Children who had received systemic corticosteroids, bronchodilators or leukotriene-receptor antagonists within the previous two weeks were also excluded in order to minimize potential confounding effects of recent medication use on respiratory symptoms and disease progression. We also excluded children whose medical record was missing > 40% of the data required for primary outcomes or key covariates. All clinical data were extracted from the hospital's electronic medical record system, and the >40% threshold referred to the proportion of missing values among variables required for the primary outcomes and key covariates within an individual patient record. A patient flow diagram illustrating the number of hospitalized children, PCR testing, exclusions, and the final cohort is provided in Figure 1 to clarify patient selection and reduce potential concerns regarding selection bias.

This study was conducted in accordance with the Declaration of Helsinki. The study protocol was reviewed and approved by the Institutional Review Board (IRB) of the First Affiliated Hospital of Ningbo University (Approval No. 173RS-01, approved in 2025). As this was a retrospective study based on existing medical records, the requirement for written informed consent was waived by the IRB.

### Data collection

A comprehensive set of variables was extracted from the electronic medical records for analysis. These included demographic characteristics such as age, sex, and having at least one sibling. Past medical history comprised premature delivery, recurrent respiratory infection, congenital heart disease (CHD), asthma, immune deficiency, inherited metabolic disorders.

Presenting symptoms reported prior to hospital admission included cough, history of wheezing, chest pain, dyspnea, hemoptysis, chills, headache, emesis, and abdominal pain. Auscultatory findings on physical examination at admission included moist crackles, wheezing on auscultation (21, 22), and diminished breath sounds. Additionally, total symptom duration prior to admission was recorded. Body temperature category was classified as normal (<37.3°C), low-grade fever (37.3–38.0°C), moderate fever (38.1–39.0°C), and ultra-hyperpyrexia (≥40.0°C). Fever duration and total symptom duration were also recorded.

All laboratory and immune function assessments were performed within 24 h of hospital admission. Laboratory indicators included white blood cell count (WBC), neutrophil percentage (N%), lymphocyte percentage (L%), hemoglobin (Hb), platelet count (PLT), C-reactive protein (CRP), erythrocyte sedimentation rate (ESR), procalcitonin (PCT), and multivarious detection. Immune function was assessed through immunoglobulin G (IgG), immunoglobulin A (IgA), immunoglobulin M (IgM), immunoglobulin E (IgE), B-cell count (CD19), total T-cell count (CD3), helper T-cell count (CD4), cytotoxic T-cell count (CD8), natural killer (NK) cell count, and CD4/CD8 ratio, plus cytokines interleukin-2 (IL-2), interleukin-4 (IL-4), interleukin-6 (IL-6), interleukin-10 (IL-10), tumor necrosis factor-alpha (TNF-α), interferon-gamma (IFN-γ). Furthermore, liver, kidney, cardiac, and coagulation functions were evaluated using parameters such as total protein, albumin, alanine aminotransferase (ALT), aspartate aminotransferase (AST), lactate dehydrogenase (LDH), creatine kinase-MB (CK-MB), prothrombin time (PT), activated partial thromboplastin time (APTT), fibrinogen (FIB), and D-dimer (D2). In our institution, an extended panel of immune and cytokine assays is routinely performed for all hospitalized children with persistent fever or suspected severe respiratory infection as part of the standard clinical evaluation protocol. Therefore, these data were available for all included patients and were not selectively measured for research purposes. Finally, treatment history included the use of antibiotics.

### Bronchitis and pneumonia

The primary outcomes of this study were the progression of HRV infection to bronchitis or pneumonia. A diagnosis of bronchitis was primarily based on the physician's clinical assessment, documented in the medical record as “bronchitis”, and supported by characteristic symptoms and chest radiography findings (1, 16). A medical record of bronchitis was supported by compatible clinical features, including cough, wheezing, or abnormal auscultatory findings (e.g., wheezing or crackles). Cases with radiographic evidence of pulmonary infiltration were classified as pneumonia and excluded from the bronchitis group. Although not all hospitalized children underwent chest imaging, the likelihood of missing lower respiratory tract involvement in this inpatient cohort was very low, as patients with atypical or severe symptoms routinely received x-ray evaluation. Pneumonia was confirmed by imaging findings, specifically a chest x-ray or CT scan, which demonstrated the presence of at least one patchy, interstitial, or lobar infiltrate consistent with parenchymal infection (23, 24). Among patients diagnosed with pneumonia, the majority underwent chest x-ray, while a subset had CT scans based on clinical indications.

### Statistical analysis

Variable selection was conducted through a two-stage machine-learning pipeline designed to distil the most informative predictors from an initial pool of 61 candidate variables (25, 26). In the first stage, a Random Forest model was fitted with 1,000 trees, and the predictive contribution of each variable was quantified by Mean Decrease Accuracy and Mean Decrease Gini indices (27). Only features that ranked within the top 50% on both importance measures were carried forward, thereby eliminating noise variables that showed negligible marginal gain in classification performance. The second stage applied Least Absolute Shrinkage and Selection Operator (LASSO) regression to the reduced feature set; ten-fold cross-validation was used to select the optimal penalty parameter (lambda.min) that minimised over-fitting while preserving generalizability (28). Coefficients that were shrunk to exact zero were interpreted as absence of any independent predictive signal, and the remaining non-zero terms were retained as the final, parsimonious signature of risk factors for disease progression.

These selected variables were subsequently entered into a multinomial logistic regression model. The reference group was no progression. We calculated odds ratios (OR) and 95% confidence intervals to see how each variable influenced the chance of developing bronchitis or pneumonia.

All statistical analyses were performed using R software (version 4.1.2). *P* < 0.05 was set as the threshold for statistical significance.

## Results

### Baseline characteristics

Of 1,125 rhinovirus-infected inpatients, 125 (11%) remained free of lower-airway disease, 332 (29%) developed bronchitis and 668 (59%) progressed to pneumonia (Table 1). The pneumonia group was older on average than the bronchitis group (3.2 vs. 2.9 years) but younger than the no-progression group (4.4 years, *p* < 0.001). They also showed the most severe respiratory illness: cough 99%, moist-rales score 0.65 ± 0.48 and symptom duration 7.4 ± 14.6 days (all *p* < 0.001). Viral co-infection (39.7%), platelets (343 ± 110 × 10⁹/L), IgM (1.21 ± 0.56 g/L) and pre-admission antibiotics (58% vs. 43%) were all higher in the pneumonia group (*p* < 0.05). Other markers did not differ.

**Table 1:** Baseline characteristics of hospitalized children with rhinovirus infection stratified by disease outcome (no progression, bronchitis, and pneumonia).

Characteristics mean (SD)	No progression (*n* = 125)	Bronchitis (*n* = 332)	Pneumonia (*n* = 668)	*P*
Age	4.41 (3.51)	2.88 (2.52)	3.24 (2.78)	<0.001
Sex (%)				
Male	78 (62.4)	220 (66.3)	400 (59.9)	0.146
Female	47 (37.6)	112 (33.7)	268 (40.1)	
Hava at least one sibling (%)	55 (44.0)	146 (44.0)	368 (55.1)	0.001
Past medical history (%)				
Premature delivery	8 (6.4)	26 (7.8)	81 (12.1)	0.035
Recurrent respiratory infection	12 (9.6)	28 (8.4)	72 (10.8)	0.502
Congenital heart disease	10 (8.0)	19 (5.7)	41 (6.1)	0.661
Asthma	11 (8.8)	65 (19.6)	104 (15.6)	0.018
Immune deficiency	0 (0.0)	0 (0.0)	7 (1.0)	0.09
Inherited metabolic disorders	4 (3.2)	7 (2.1)	22 (3.3)	0.569
History of wheezing	10 (8.0)	73 (22.0)	143 (21.4)	0.002
Body temperature (%)				
Normal	22 (17.6)	13 (3.9)	5 (0.7)	0.002
Low/moderate fever	29 (23.2)	117 (35.5)	207 (31.1)	
Ultra-hyperpyrexia	60 (48.0)	93 (28.2)	214 (32.1)	
Fever duration, day	2.06 (2.68)	1.65 (2.56)	2.21 (3.20)	0.019
Symptom (%)				
Cough	103 (82.4)	319 (96.1)	663 (99.3)	<0.001
History of wheezing	0.22 (0.41)	0.54 (0.50)	0.55 (0.50)	<0.001
Chest pain	6 (4.8)	3 (0.9)	8 (1.2)	0.006
Dyspnea	0.11 (0.32)	0.44 (0.50)	0.38 (0.49)	<0.001
Hemoptysis	0 (0.0)	0 (0.0)	3 (0.4)	0.357
Chill	0.02 (0.15)	0.02 (0.14)	0.02 (0.13)	0.879
Headache	0.06 (0.23)	0.02 (0.12)	0.01 (0.11)	0.003
Emesis	29 (23.2)	69 (20.8)	133 (19.9)	0.699
Abdominal pain	0.07 (0.26)	0.06 (0.23)	0.04 (0.19)	0.144
Moist rales	0.20 (0.40)	0.47 (0.50)	0.65 (0.48)	<0.001
Wheezing on auscultation	0.17 (0.38)	0.53 (0.50)	0.51 (0.50)	<0.001
Diminished breath sounds	6 (4.8)	0 (0.0)	29 (4.3)	0.001
Symptom duration, day	5.07 (5.45)	4.39 (5.04)	7.44 (14.59)	<0.001
WBC	11.58 (6.20)	18.25 (97.35)	12.15 (5.33)	0.229
Neutrophil count (%)	58.51 (18.01)	58.18 (22.10)	57.79 (20.63)	0.927
Lymphocyte count (%)	34.30 (27.99)	32.66 (20.26)	32.90 (18.80)	0.765
Hb (g/L)	123.25 (12.47)	123.17 (12.65)	125.24 (21.89)	0.224
PLT	305.71 (118.08)	331.38 (121.51)	342.78 (110.48)	0.006
CRP	17.57 (31.56)	17.00 (35.48)	16.16 (32.29)	0.882
ESR (mm/h)	19.08 (21.35)	20.93 (20.25)	20.98 (19.10)	0.713
PCT (ng/mL)	0.41 (1.06)	0.49 (3.81)	0.41 (2.25)	0.912
Viral co-infection (%)	31 (24.8)	69 (20.8)	265 (39.7)	<0.001
Total protein	67.52 (5.09)	67.31 (6.77)	67.54 (6.28)	0.863
Albumin	41.46 (3.62)	42.39 (4.16)	42.14 (4.09)	0.104
ALT	26.93 (80.76)	24.52 (39.74)	20.74 (17.12)	0.119
AST	47.38 (58.72)	45.27 (34.59)	42.65 (26.09)	0.257
LDH	386.10 (208.84)	376.18 (221.52)	379.52 (230.83)	0.919
CK-MB	41.22 (53.27)	35.35 (30.25)	34.40 (30.23)	0.126
IgG (g/L)	8.58 (2.87)	8.33 (2.90)	8.37 (2.94)	0.815
IgA (g/L)	0.94 (0.72)	0.81 (0.70)	0.81 (0.69)	0.338
IgM (g/L)	1.04 (0.50)	1.11 (0.48)	1.21 (0.56)	0.008
IgE (IU/mL)	182.61 (258.07)	282.09 (344.53)	245.84 (327.58)	0.079
IL-2	2.01 (0.86)	2.07 (0.81)	2.00 (1.00)	0.755
IL-4	2.40 (0.76)	2.49 (0.74)	2.52 (1.09)	0.719
IL-6	161.29 (433.50)	126.47 (278.64)	120.71 (449.81)	0.804
IL-10	12.15 (13.71)	15.64 (41.52)	11.89 (13.28)	0.38
TNF	3.06 (3.32)	2.89 (2.67)	2.75 (3.90)	0.821
IFN	5.10 (8.89)	5.75 (9.42)	8.48 (30.32)	0.448
CD19	20.99 (9.10)	24.32 (12.00)	25.19 (11.14)	0.134
CD3	68.21 (10.82)	63.34 (14.38)	65.47 (11.08)	0.128
CD4	35.88 (9.17)	33.25 (11.59)	35.62 (10.22)	0.227
CD8	24.22 (7.58)	23.24 (11.02)	22.69 (8.21)	0.651
NK	10.38 (6.66)	9.76 (5.49)	9.45 (6.30)	0.711
CD4/CD8	2.24 (3.36)	1.82 (1.55)	1.77 (0.85)	0.303
PT (s)	11.70 (1.08)	11.72 (1.02)	11.76 (1.08)	0.859
FIB (g/L)	3.48 (1.51)	3.34 (1.25)	3.34 (1.31)	0.68
APTT (s)	28.27 (3.50)	28.14 (4.00)	27.88 (3.50)	0.57
D2	1.17 (3.34)	0.64 (1.55)	0.63 (1.59)	0.062
Use antibiotics before admission (%)	34 (48.6)	100 (42.7)	208 (58.3)	0.001

WBC, white blood cell count; N%, neutrophil percentage; L%, lymphocyte percentage; Hb, hemoglobin; PLT, platelet count; CRP, C-reactive protein; ESR, erythrocyte sedimentation rate; PCT, procalcitonin; IgG, immunoglobulin G; IgA, immunoglobulin A; IgM, immunoglobulin M; IgE, immunoglobulin E; CD19, B-cell count; CD3, total T-cell count; CD4, helper T-cell count; CD8, cytotoxic T-cell count; NK, natural killer cell count; CD4/CD8 ratio; IL-2, interleukin-2; IL-4, interleukin-4; IL-6, interleukin-6; IL-10, interleukin-10; TNF-α, tumor necrosis factor-alpha; IFN-γ, interferon-gamma; ALT, alanine aminotransferase; AST, aspartate aminotransferase; LDH, lactate dehydrogenase; CK-MB, creatine kinase-MB; PT, prothrombin time; APTT, activated partial thromboplastin time; FIB, fibrinogen; D2, D-dimer.

### Variable selection

**Figure 2:**
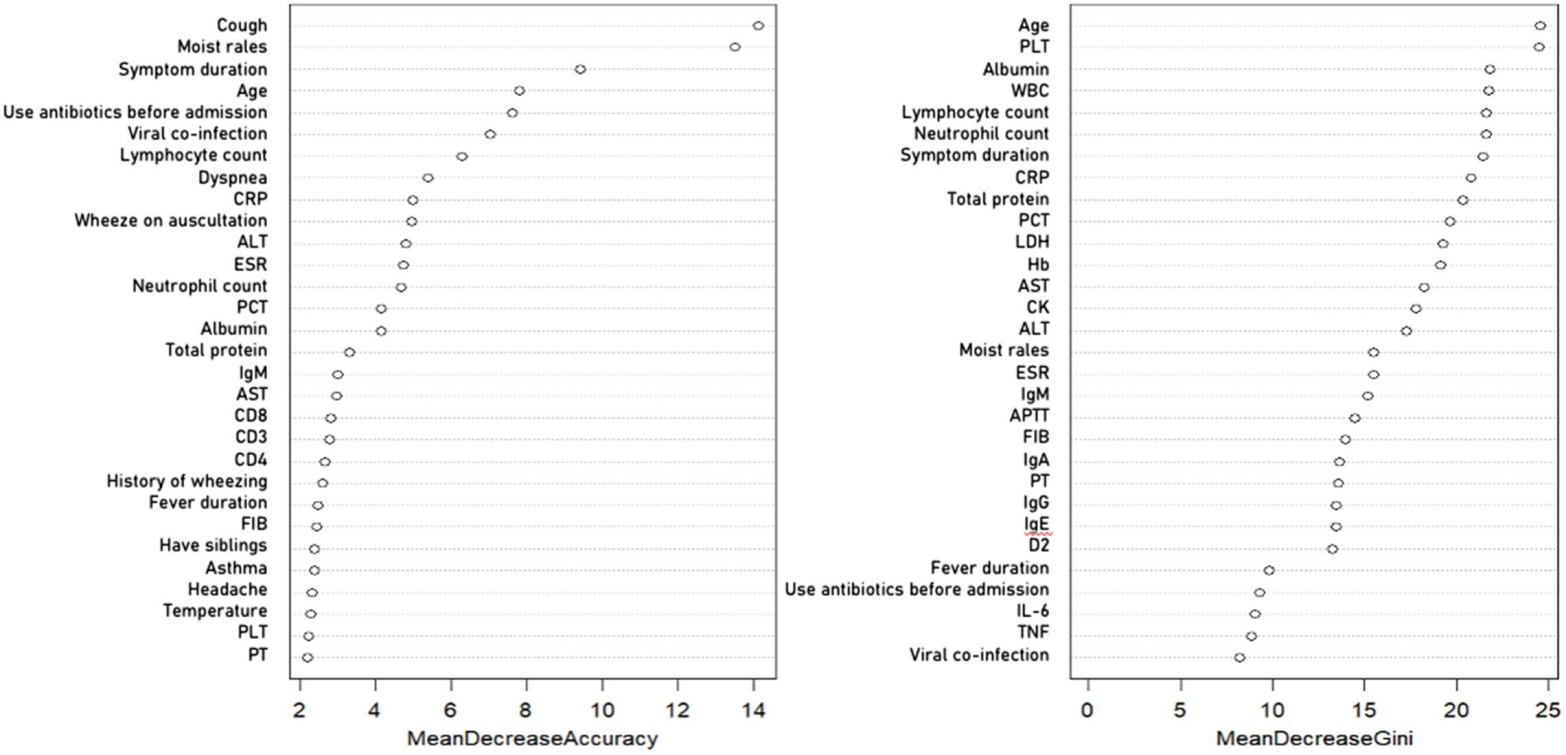
Variable importance derived from the random forest model for predicting progression to bronchitis or pneumonia among hospitalized children with rhinovirus infection. Hb, hemoglobin; PLT, platelet count; CRP, C-reactive protein; ESR, erythrocyte sedimentation rate; PCT, procalcitonin; IgA, immunoglobulin A; IgM, immunoglobulin M; IgE, immunoglobulin E; CD3, total T-cell count; CD4, helper T-cell count; CD8, cytotoxic T-cell count; IL-6, interleukin-6; ALT, alanine aminotransferase; AST, aspartate aminotransferase; PT, prothrombin time; APTT, activated partial thromboplastin time; FIB, fibrinogen; D2, D-dimer.

The variable importance analysis by random Forest model screened and retained 31 variables from 61 independent variables (Figure 2). These included clinical symptom variables such as cough, moist rales, dyspnea, wheezing on auscultation, history of wheezing, fever duration, headache, body temperature records, and symptom duration. Laboratory indicators comprised white blood cell differentials, inflammatory markers including C-reactive protein and erythrocyte sedimentation rate, liver function tests such as aspartate aminotransferase and alanine aminotransferase, immune cell subsets including CD3, CD4, CD8, and CD19, plasma proteins such as albumin and total protein, immunoglobulin M, and platelet count. Demographic and medical history variables encompassed age, multivirus infection, having at least one sibling, history of asthma, and antibiotic use before admission. Their mean decrease accuracy and mean decrease gini scores were significantly higher than those of other indicators.

**Figure 3:**
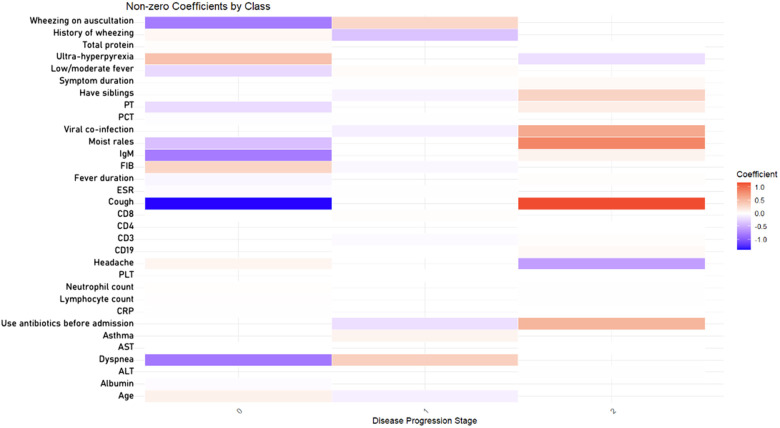
Heatmap of variable coefficients associated with disease progression (bronchitis and pneumonia) in children with rhinovirus infection. The color intensity represents the magnitude of coefficients, with red indicating a positive association and blue indicating a negative association. PLT, platelet count; CRP, C-reactive protein; ESR, erythrocyte sedimentation rate; PCT, procalcitonin; IgM, immunoglobulin M; CD3, total T-cell count; CD4, helper T-cell count; CD8, cytotoxic T-cell count; ALT, alanine aminotransferase; AST, aspartate aminotransferase; PT, prothrombin time; APTT, activated partial thromboplastin time; FIB, fibrinogen.

**Figure 4:**
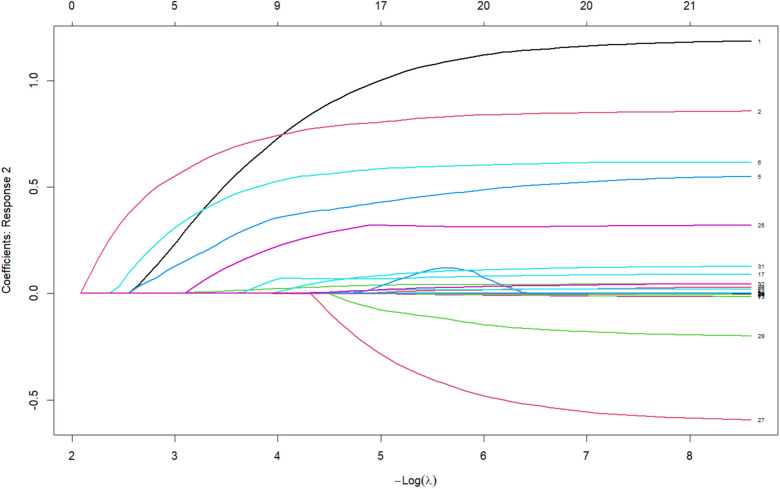
Coefficient path plot of LASSO regression used for variable selection in modeling disease progression among children with rhinovirus infection.

The variables selected by the Random Forest model were further subjected to LASSO regression for refined screening. Figure 3 displays a heatmap across three outcomes: no progression (class 0), bronchitis (class 1), and pneumonia (class 2). Key predictors included cough (β = −1.39 for class 0; β = 1.17 for classes 1 and 2), dyspnea (β = −0.81 for class 0; β = 1.17 for class 2), and IgM (β = −0.79 for class 0; β = 1.17 for class 2). Age (β = 0.10) and fibrinogen (β = 0.31) were positively associated with pneumonia. The coefficient path plot (Figure 4) shows that 21 variables were retained. The final set of predictors included age, have siblings, asthma history, use of antibiotics before admission, cough, history of wheezing, headache, low/moderate fever, ultra-hyperpyrexia, fever duration, overall symptom duration, moist rales, wheezing on auscultation, dyspnea, viral co-infection, FIB, CD3, CD19, IgM, and albumin.

### Logistic regression

**Figure 5:**
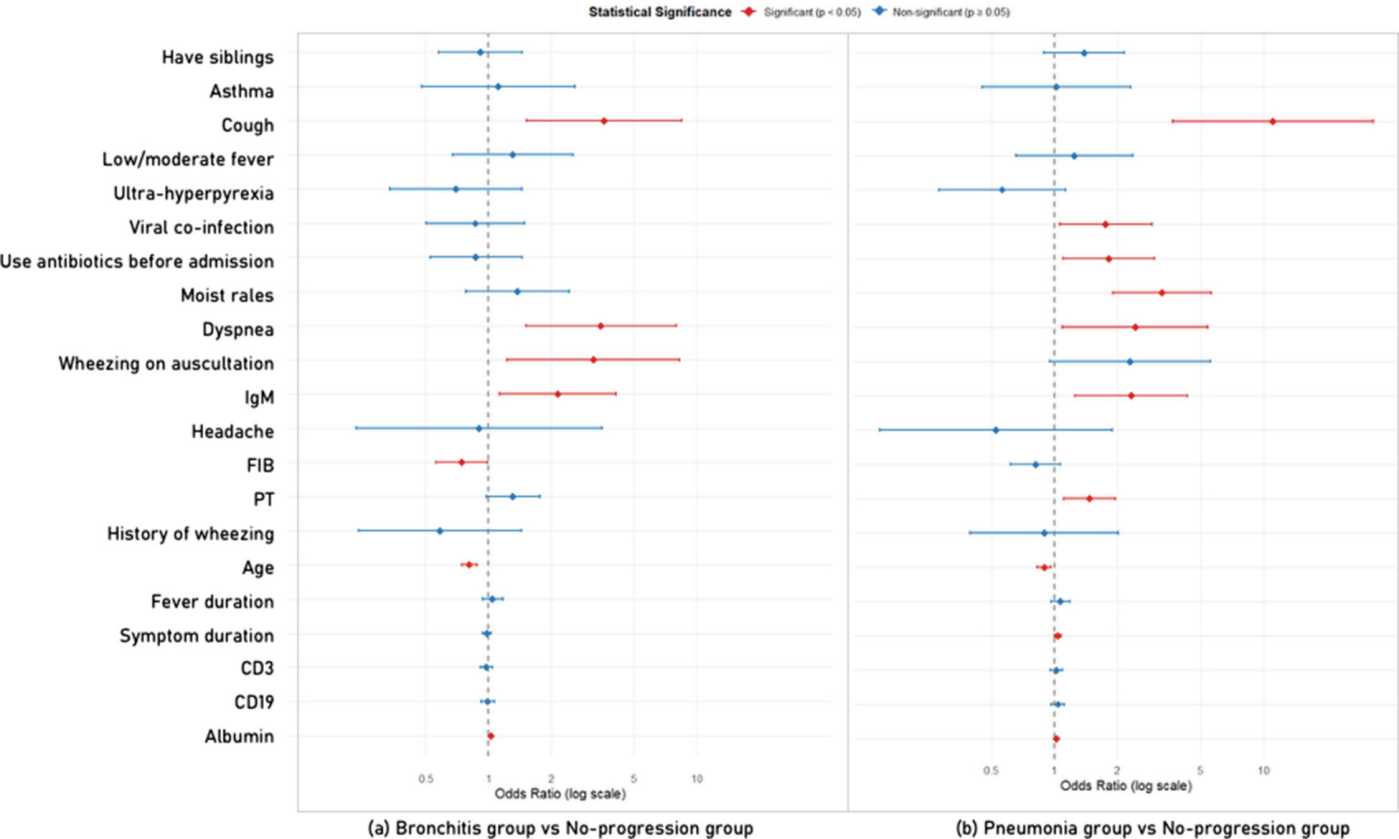
Multinomial logistic regression analysis of factors associated with bronchitis and pneumonia (reference: no progression) in children with rhinovirus infection. FIB, fibrinogen. PT, prothrombin time.

The selected variables were incorporated into a multinomial logistic regression analysis, and the results are presented in Figure 5. When comparing the bronchitis group with the no-progression group, the model identified several key predictive factors. dyspnea (*OR* = 3.43, 95% *CI*: 1.49–7.90), wheezing on auscultation (*OR* = 3.07, 95% *CI*: 1.18–7.99), and cough (*OR* = 3.24, 95% *CI*: 1.42–7.40) showed the strongest associations. Elevated IgM levels (*OR* = 2.23, 95% *CI*: 1.17–4.25) were also linked to higher risk. Older age was associated with lower risk (*OR* = 0.81, 95% *CI*: 0.74–0.88).

In the comparison between the pneumonia group and the no-progression group, the predictive factor profile differed. Cough showed the strongest association with higher risk (*OR* = 10.46, 95% *CI*: 3.55–30.80), followed by moist rales (*OR* = 3.30, 95% *CI*: 1.93–5.64). Other factors linked to higher risk included elevated IgM (*OR* = 2.42, 95% *CI*: 1.30–4.49), prolonged prothrombin time (*OR* = 1.41, 95% *CI*: 1.10–1.80), prior antibiotic use (*OR* = 1.88, 95% *CI*: 1.14–3.10), and viral co-infection (*OR* = 1.68, 95% *CI*: 1.01–2.78). Longer symptom duration was also associated with higher risk (*OR* = 1.04, 95% *CI*: 1.00–1.08), while older age was associated with lower risk (*OR* = 0.89, 95% *CI*: 0.83–0.96).

## Discussion

This study identified key risk factors associated with the progression of rhinovirus infection to specifically bronchitis or pneumonia, in hospitalized children using a two-stage machine learning strategy. Then we included these factors into multinomial logistic regression analysis to identify distinct and robust predictive profiles for bronchitis and pneumonia. Progression to bronchitis was primarily driven by lower airway obstructive signs (dyspnea, wheezing, cough). Conversely, progression to pneumonia was most strongly associated with cough and moist rales, but uniquely influenced by factors indicating a more complex clinical picture, such as prior antibiotic use, viral co-infection, and coagulation abnormalities. Notably, elevated IgM was a common risk factor for both conditions, while older age was consistently protective. These findings highlight that different pathological pathways underlie the progression to bronchitis vs. pneumonia, enabling more precise early identification of patients at high risk for severe outcomes.

The distinct risk factor profiles identified for bronchitis and pneumonia progression expand upon previous research on HRV-associated LRTIs in pediatric populations. Prior studies have consistently linked wheezing and lower airway obstruction to HRV-induced bronchitis exacerbations, particularly in children with underlying asthma or bronchial hyperresponsiveness (29, 30). Our finding that dyspnea, wheezing on auscultation, and cough are primary drivers of bronchitis progression reinforces the notion that HRV-induced airway inflammation directly precipitates obstructive lower airway pathology (31). In contrast, the association of pneumonia with prior antibiotic use, viral co-infection, and coagulation abnormalities sheds new light on the multifactorial pathways leading to parenchymal lung involvement. Prior antibiotic exposure may disrupt the airway microbiome's protective role and increase the susceptibility to secondary bacterial infection or enhancing HRV ability to invade alveolar epithelial cells (32). Viral co-infection may have a synergistic pathogenic effect, exacerbating respiratory mucosal damage and inflammatory responses, thereby increasing the risk of progression to pneumonia (33).

The observation that elevated IgM is a shared risk factor for both bronchitis and pneumonia warrants further mechanistic exploration. As an antibody produced early in infection, increased IgM levels serve as serological evidence of acute infection with rhinovirus or other co-infecting pathogens (34). This suggests that recent immune activation is a key mechanism driving progression to LRTIs (35). Notably, traditional inflammatory markers like CRP and PCT did not show strong predictive power (36, 37). The reason may relate to the nature of viral infections that their levels usually rise less than in bacterial infections (38). Conversely, the protective effect of older age is consistent with epidemiological data showing that infants and young toddlers have immature immune systems and underdeveloped airway structures, rendering them more vulnerable to severe RV-associated LRTIs (39). This age-related protection likely reflects the gradual maturation of mucosal immunity, enhanced viral clearance capabilities, and reduced susceptibility to airway obstruction with increasing age.

A promising direction of this study for future research is the development of digital auscultation technologies. Traditional stethoscope-based assessment is inherently subjective and may vary across clinicians, which can affect the consistency of lung sound interpretation. Recent advances in digital stethoscopes and electronic auscultation platforms allow respiratory sounds to be recorded, shared, and analyzed computationally, facilitating more standardized evaluation of lung sounds (40, 41). In addition, artificial intelligence–assisted algorithms have demonstrated promising performance in detecting wheezing and other respiratory sounds in pediatric populations (42). Integration of these technologies into clinical research and practice may improve the objectivity, reproducibility, and scalability of respiratory sound assessment in future studies.

## Limitations

This study has several limitations. First, the study was conducted in a single hospital setting, which may limit the generalizability of the risk factor profiles to other pediatric populations. Future study could expand the population to outpatient children or those in low-resource settings. Second, the study lacked long-term outcomes (e.g., asthma development, recurrent LRTIs, mortality) in children with HRV-associated bronchitis or pneumonia. Last, the potential for residual confounding exists due to the inability to adjust for key unmeasured variables, such as nutritional status, vaccination history, and environmental exposures.

## Conclusion

In summary, this study identifies distinct risk factor profiles for the progression of RV infection to bronchitis or pneumonia in hospitalized children. Bronchitis progression in these children is mainly driven by lower airway obstructive signs: dyspnea, wheezing on auscultation, and cough. In contrast, pneumonia is most linked to cough, moist rales, prior antibiotic use, viral co-infection, and coagulation abnormalities. Notably, elevated IgM is a common risk factor for both conditions, while older age protects against both. These findings hold significant clinical utility for improving early risk stratification and targeted intervention in hospitalized children with RV infection.

## Data Availability

The raw data supporting the conclusions of this article will be made available by the authors, without undue reservation.
